# Supplementary material for: Older adults’ suggestions of research topics on ageing well in urban environments – A participatory study
Source: PLoS One. 2023 Oct 4;18(10):e0292214. doi: 10.1371/journal.pone.0292214 (PMC10550106; doi:10.1371/journal.pone.0292214)
Supplement: Attachment — Submitted filename: response_to_reviewers.docx [file pone.0292214.s001.docx]

Dear editor and reviewers,

Thank you very much for considering our manuscript and taking the time to give valuable comments. We implemented all your revisions and updated the manuscript accordingly. Furthermore, we corrected a small error in the number of participants that we found during revision. We now think that the manuscript has greatly improved and hope that it is acceptable for publication. Below, we reply to your comments point by point.

**EDITOR**

*1. Please ensure that your manuscript meets PLOS ONE's style requirements, including those for file naming. The PLOS ONE style templates can be found at ...*

We checked the manuscript to meet PLOS ONE’s style requirements according to your templates and updated the file names prior to uploading the revision.

*2. Please provide additional details regarding participant consent. In the ethics statement in the Methods and online submission information, please ensure that you have specified what type you obtained (for instance, written or verbal, and if verbal, how it was documented and witnessed). If your study included minors, state whether you obtained consent from parents or guardians. If the need for consent was waived by the ethics committee, please include this information."*

Participants were asked to answer the questionnaire anonymously and were informed that by answering, implied consent was given to participate and for publication of the results. We updated the methods section and the online submission information accordingly.

*3. Our internal editors have looked over your manuscript and determined that it is within the scope of our Aging in Human Health and Disease Call for Papers. […]*

We are grateful that our manuscript is being considered for the Aging in Human Health and Disease Call for Papers and that we can contribute to the collection.

*4. Please review your reference list to ensure that it is complete and correct. If you have cited papers that have been retracted, please include the rationale for doing so in the manuscript text, or remove these references and replace them with relevant current references. Any changes to the reference list should be mentioned in the rebuttal letter that accompanies your revised manuscript.*

We made three changes to the reference list: we updated the format of reference no. 1 and no. 2 and added URLS to the respective databases. Also, reference no. 13 (Neugarten BL. Age Groups in American Society and the Rise of the Young-Old) was added.

*While revising your submission, please upload your figure files to the Preflight Analysis and Conversion Engine (PACE) digital diagnostic tool,* [*https://pacev2.apexcovantage.com/*](https://pacev2.apexcovantage.com/)*.*

The figure we use in the manuscript is now processed by PACE and will be re-uploaded in the new format.

**Additional Editor Comments (if provided):**
*Your topic is excellent and significant and the methods appropriate. Please submit a minor revision - I suggest you consult an English speaking helper to improve a few passages in the writing that may be improved for clarity and fluency.*

We have revised the manuscript together with an English speaking colleague to improve text flow and clarity.

---

**REVIEWER 1**
*Please update quotation marks to standard US English formatting.*

Thank you for your revisions on the manuscript. As part of the language editing for this revision, we corrected the passages in which we are using quotation marks to fit the US English formatting.

*Check the use of "sealing" and "seating" on lines 169 and 171. Are these supposed to be the same word?*

The first quote mentioning “sealing” refers to the sealing of soil in urban green spaces, while the second quote “seating” refers to seating possibilities. We changed the wording in the manuscript to make the difference clearer (lines 179-181 in the manuscript with mark-ups).

---
**REVIEWER 2**
*The idea of looking at "older adults health needs" is very valuable, but it seems that the inclusion of all participants in the age brackets (65 +) can be confusing. Authors should clarify if they are combining all "older adults"? If so, this can be problematic because the needs of this population and as such research needs can vary according to the specific chronological ages. That is a 65-year-old person's needs can be quite different than an eighty-year-old, although there could be important similarities. Clarification of the cohort groups and what are the common characteristics they share is required. Additionally, and because of these different characteristics of the cohort groups, need to be defined more clearly. It is recommended that the authors strengthen the categories with a clearer definition of the different ages and specific needs.*

Thank you very much for this important comment. Due to data security reasons, we decided not to ask for additional demographic data of the participants, such as age and socioeconomic status to keep the data anonymous. We acknowledge that the needs and research ideas of different age groups can be quite different. Thus, we added a paragraph in the limitations sections of the manuscript describing this inherent limitation of our study.

Kind regards,

Philip Oeser on behalf of all the authors
